# Central Auditory Tests to Track Cognitive Function in People With HIV: Longitudinal Cohort Study

**DOI:** 10.2196/26406

**Published:** 2021-02-09

**Authors:** Christopher Niemczak, Abigail Fellows, Jonathan Lichtenstein, Travis White-Schwoch, Albert Magohe, Jiang Gui, Jed Wilbur, Odile Clavier, Enica Massawe, Ndeserua Moshi, Michael Boivin, Nina Kraus, Jay Buckey

**Affiliations:** 1 Geisel School of Medicine at Dartmouth Dartmouth College Lebanon, NH United States; 2 Dartmouth-Hitchcock Medical Center Lebanon, NH United States; 3 Department of Communication Sciences and Disorders Northwestern University Chicago, IL United States; 4 Dar Dar Programs Dar es Salaam United Republic of Tanzania; 5 Creare LLC Hanover, NH United States; 6 Muhimbili University of Health and Allied Sciences Dar es Salaam United Republic of Tanzania; 7 Department of Psychiatry Michigan State University East Lansing, MI United States

**Keywords:** HIV, central auditory function, auditory perception, cognitive dysfunction, testing, cognition, cognitive function, neurocognitive deficit, longitudinal, auditory, nervous system, screening, monitoring, surveillance

## Abstract

**Background:**

The development of neurocognitive deficits in people infected with HIV is a significant public health problem. Previous cross-sectional studies have shown that performance on central auditory tests (CATs) correlates with cognitive test results in those with HIV, but no longitudinal data exist for confirmation. We have been performing longitudinal assessments of central auditory and cognitive function on a cohort of HIV-positive and HIV-negative individuals in Dar es Salaam, Tanzania to understand how the central auditory system could be used to study and track the progress of central nervous system dysfunction.

**Objective:**

The goal of the project was to determine if CATs can track the trajectory of cognitive function over time in people diagnosed with HIV.

**Methods:**

Tests of peripheral and central auditory function as well as cognitive performance were performed on 382 individuals over the course of 3.5 years. Visits were scheduled every 6 months. CATs included tests of auditory temporal processing (gap detection) and speech perception in noise (Hearing in Noise Test and Triple Digit Test). Cognitive tests included the Montreal Cognitive Assessment (MoCA), Test of Variables of Attention (TOVA), and subtests from the Cogstate battery. HIV-positive subjects were divided into groups based on their CAT results at their final visit (bottom 20%, top 20%, middle 60%). Primary analyses focused on the comparison between HIV-positive individuals that performed worse on CATs (bottom 20%) and the overall HIV-positive group (middle 60%). Data were analyzed using linear mixed-effect models with time as the main fixed effect.

**Results:**

The group with the worst (bottom 20%) CAT performance showed a difference in trajectory for the MoCA (*P*=.003), TOVA (*P*<.048), and Cogstate (*P*<.046) over the course of the study period compared to the overall HIV-positive group. A battery of three CATs showed a significant difference in cognitive trajectory over a relatively short study period of 3.5 years independent of age (bottom 20% vs HIV-positive group).

**Conclusions:**

The results of this study support the ability for CATs to track cognitive function over time, suggesting that central auditory processing can provide a window into central nervous system performance. CATs can be simple to perform, and are relatively insensitive to education and socioeconomic status because they only require repeating sentences, numbers, or detecting gaps in noise. These tests could potentially provide a time-efficient, low-cost method to screen for and monitor cognitive decline in patients with HIV, making them a useful surveillance tool for this major public health problem.

## Introduction

Even with advanced antiretroviral therapy, people infected with HIV can develop neurocognitive deficits [1]. This consequence of HIV infection produces a lifelong reduction in quality of life and poses a major public health concern. The ability to track HIV-associated central nervous system (CNS) effects is critical for studying, assessing, and treating this serious complication of HIV infection. However, detecting emergent neurocognitive problems is challenging, particularly in the developing world where most HIV cases exist. Neurocognitive test batteries can take considerable time to administer (approximately 2 hours for the National Institutes of Health toolbox); require trained personnel; and depend on accurate, culturally/linguistically appropriate normative data for interpretation. Deploying these tests is difficult, particularly in the developing world where clinician time is limited, few trained personnel are available, and normative data often do not exist. Improved surveillance methods for cognitive decline are needed, particularly in those with HIV.

We have been examining the use of central auditory tests (CATs) as an approach for tracking cognitive function in HIV-positive individuals. Our earlier work established that performance on CATs in HIV-positive individuals is strongly related to cognitive test results [2]. This suggests that central auditory testing evaluates aspects of brain function, and could potentially be used to screen and monitor neurocognitive dysfunction in HIV-positive individuals. However, to date, there has not been a longitudinal study of CATs and cognitive function in HIV-positive individuals. Therefore, the goal of this study was to track cognitive and CAT results over time, and examine the relationship between the two measures.

The central auditory system provides a window into brain function because processing complex auditory information is a neurologically demanding task [3,4]. After the cochlea converts sound waves into nerve signals, the brain must quickly filter out noise, extract timing information, distinguish relevant frequencies within the signals, and determine the meaning of the content. This involves neural pathways throughout the brainstem and into the cortex that integrate with linguistic and cognitive systems, which depend on adequate processing speed, working memory, and attention [5-7]. In addition, previous studies have shown that HIV-positive individuals develop signs of central auditory processing that cannot be attributed to peripheral hearing loss [4,8,9]. Even with normal peripheral hearing sensitivity (eg, normal pure-tone auditory threshold test results), HIV-positive individuals show degraded results on tests of speech perception in noise [10] and auditory gap detection [4].

Problems with central auditory processing often manifest as difficulty understanding speech, particularly in the context of background noise. Accurate speech perception requires complex processing in the auditory midbrain and cortex [7,11,12]. Speech perception in background noise challenges the listener and stresses the central auditory system, requiring the listener to attend to the speech signal, match what is heard to stored knowledge, and derive meaning [13-16]. Most people can attest that understanding a conversational partner in a crowded, noisy room is a common yet difficult task, even for those with normal hearing. This process takes place within milliseconds, and requires high-level cognitive functions such as working memory and executive function [17,18].

To test central auditory processing, we have assembled a battery of behavioral CATs that measure the system in two critical ways: temporal auditory processing and speech perception in noise. Temporal processing refers to the precise perception of time alterations on audible acoustic events [19]. Deficits in temporal processing have been associated with attention problems [20,21] and overall difficulty encoding brief relevant auditory stimuli needed for accurate speech perception [22]. Speech perception in background noise is a broader functional test that involves listeners attending to the auditory signal within noise, performing acoustic analysis, mapping the signal to phonemic categories, temporarily storing acoustic information in memory for further processing, and finally mapping phonemes to meaning [15]. Cognitive factors such as attention, working memory, and speed of processing contribute significantly to both speech perception in quiet and in noise [14,23]. For example, Humes [24] found that part of the variance in speech recognition in noise can be accounted for by nonperipheral factors, including cognitive functions. Using structural equation modeling, Anderson et al [25] showed a strong influence of cognitive factors on speech-in-noise perception, whereas peripheral hearing ability was not a significant contributor.

CATs have several practical advantages over cognitive assessments: they do not require literacy or a high level of education to complete; they are short and easy to explain; and some tests can even be administered remotely, by phone or internet. Thus, these tests could be a major advance for following HIV-positive patients, particularly in the developing world. If performance on CATs can track or provide an early marker of CNS dysfunction in HIV infection, detecting these changes in clinical practice could lead to appropriate adjustments in HIV treatment. CATs could be used to identify CNS comorbidities or to track treatment effects. Antiretroviral drugs differ in their ability to penetrate the CNS to treat HIV, and resistance to particular antiretrovirals can develop over time [26-28]. For example, a change in antiretroviral drug regimen [29], rehabilitative auditory training [30], or signal enhancement approaches [31] might prevent further deterioration in CAT performance. As degraded speech perception and overall degraded hearing have been linked with social isolation [18], it is also important to identify these individuals and assist in rehabilitation efforts as quickly as possible.

This study was designed to ascertain how longitudinal performance on CATs relates to neurocognitive performance in a cohort of HIV-negative and HIV-positive individuals. We hypothesized that the diffuse white matter disease associated with HIV infection would affect central auditory processing progressively [4,32,33]. This suggests that the effects of HIV on the CNS could be tracked with CATs, which are quantitative, time-efficient, and repeatable. If CATs have reasonable sensitivity and specificity for detecting concurrent cognitive problems, they would offer an effective surveillance metric to follow individuals with ongoing HIV infection, and could perhaps change how HIV patients are medically monitored. This would provide valuable public health information to monitor, track, and potentially predict cognitive decline due to HIV.

## Methods

### Recruitment

We recruited participants in this study from a unique cohort of approximately 670 HIV-positive and HIV-negative individuals in Dar es Salaam, Tanzania, who have been performing central auditory, peripheral auditory, and cognitive testing at approximate 6-month intervals for the last 4 years. The research protocol was approved by the Committee for the Protection of Human Subjects of Dartmouth College and the Research Ethics Committee of Muhimbili University of Health and Allied Sciences. All participants provided written informed consent.

### Study Procedures

Subjects completed a series of questionnaires, and performed cognitive and auditory tests at the Infectious Disease Center in Dar es Salaam, Tanzania. The questionnaires gathered data on the participants’ self-reported hearing ability (hearing status questionnaire) and general health (health history questionnaire). The questions covered noise exposure, tinnitus, ear drainage, ear infections, chemical exposure, and balance problems. The questionnaire also asked about past or current tuberculosis treatment; HIV treatment; gentamicin exposure; and the use of antimalarials, aspirin, and diuretics. All participants completed testing at approximately 6-month intervals; not all participants adhered to the schedule and some dropped out of the study during this time.

To ensure accuracy of longitudinal analysis, and control for variables that could affect central auditory and cognitive function tests, we used a series of data selection techniques. First, individuals were excluded if they only completed 3 or less visits. Second, data from visits beyond 3.5 years were excluded to limit bias from the subset of subjects with longer follow up (ie, a few subjects with long follow-up times could have greater leverage in the model). Third, individuals were excluded if they had abnormal hearing sensitivity (>25 dB HL from 0.5 to 4 kHz) or abnormal middle ear function. Fourth, individuals were also excluded if they had a positive history of ear drainage, concussion, significant noise or chemical exposure, neurological disease, mental illness, ototoxic antibiotics (eg, gentamycin), or chemotherapy. This selection technique resulted in a final sample of 382 individuals.

### Peripheral Auditory Tests

Peripheral auditory tests included tympanometry and audiometry after otoscopy with cerumen removal as needed to ensure a clear ear canal. A Madsen Otoflex 100 system (GN Otometrics, Denmark) was used to perform tympanometry at 226 Hz. Measurements of ear canal volume, static admittance, tympanometric peak pressure, tympanometric width, and tympanogram type (A, A_s_, A_d_, B, C) were collected. Type A tympanograms (including A_s_ and A_d_) were required for inclusion in this study, with pressure limits from –100 to +50 daPa and static admittance limits from 0.3 to 1.7 milimho.

Pure-tone air conduction thresholds were measured at frequencies of 0.5, 1.0, 2.0, and 4.0 kHz using a Békésy-like tracking procedure as previously described [33]. Pulsed tones with a duration of 250 milliseconds, a rise and fall time of 20 milliseconds, and an interstimulus interval of 500 milliseconds were used. When the button was pressed, the tone decreased in 4-dB steps until the first reversal, and then 2-dB step decreases were used. Upon releasing the button, the tones increased in 2-dB steps. A total of six good reversals were counted to identify the threshold. Normal peripheral hearing sensitivity (<25 dB HL from 0.5 to 4 kHz) was required for all subjects.

Audiometry and all behavioral audiometric testing were completed using a Creare LLC wireless automated hearing test system (WAHTS) controlled through a laptop. The WAHTS allowed for testing in rooms with minimal background noise, as the device speakers are mounted in the ear cups. The attenuation provided by this headset is on par with a portable sound booth as measured by an independent laboratory according to the relevant American National Standards Institute standards [34]. This technology provided a platform to complete high-quality audiometry and CATs in a resource-limited setting. In addition, the WAHTS included Kiswahili language versions of the CATs.

### CATs

CATs included the Hearing In Noise Test (HINT), Triple Digit Test (TDT), and gap detection test (GAP). The HINT was administered in four test conditions: noise front, noise right, noise left, and quiet. In each HINT, a different list of 20 sentences was presented in random order in the presence of the masking noise spectrally matched to the long-term average of the target material. The presentation level of the noise remained fixed at 65 dB (A-weighting), and the test instrument adjusted the level of each sentence adaptively depending on whether the test administrator indicated that the previous sentence was repeated correctly. The presentation level of the sentence was reduced if the previous sentence was repeated correctly and was increased if the previous sentence was repeated incorrectly. This adaptive procedure was used to determine the presentation level of each sentence in the list. The average presentation level of all sentences after the first four sentences defined the speech reception threshold for the test condition expressed as a signal-to-noise ratio (SNR). The WHATS displayed and recorded the SNR for each test condition. A composite SNR of all three noise conditions was calculated and used as the primary variable of interest for the HINT.

In the TDT, recordings of natural productions of three-digit triplets such as 3-5-9 (spoken as “tatu-tano-tisa” in Kiswahili) were used as target stimuli (Kiswahili numbers below 10 have the same number of syllables). All digit triplets were produced and recorded by a male speaker in a soundproof booth. Triplet digit recognition was tested in the presence of competing Schroeder-phase masking noise. The test included 30 total presentations of pseudorandom triplet digits with six practice presentations. Presentations were delivered in pairs of positive- and negative-phase maskers. Each pair was presented at the same SNR, and the order of the masker was randomized for each pair. The test started at a 0 dB initial SNR with the masker fixed at a 75 dB sound pressure level. SNRs were then adjusted after each presentation or pair of presentations by varying the target level; a 1.5 dB sound pressure level was added to the target level for each incorrect digit and a 1.5 dB sound pressure level was subtracted for each correct digit from the previous positive-phase presentation. The speech reception threshold was calculated as the SNR of the last 14 positive-phase presentations, which was used as the primary variable of interest.

We also implemented an adaptive GAP test to evaluate temporal auditory processing. The adaptive gap detection algorithm applies a single staircase and has been used extensively in our previous studies [4,33,35]. In the algorithm, the gap length is shortened when the subject correctly identifies two gaps in a row. If the subject misidentifies two gaps in a row or three gaps overall, the staircase “reverses,” and the gap length increases. In this way, the staircase algorithm converges to the subject’s gap threshold. The subjects received training in the GAP test with both a training video and a screen that provided both auditory and visual feedback. The operator presented gaps to the subject until the subject comprehended the task.

### Cognitive Tests

We used three cognitive tests: the Montreal Cognitive Assessment (MoCA), the Tests of Variables of Attention (TOVA), and selected subtests from the Cogstate battery. The MoCA was used to assess the participants’ general cognitive abilities and screen for potential cognitive impairment [36]. Questions on the MoCA focus on the areas of visual-spatial abilities (cube and clock drawing), executive function (trail making, verbal abstraction, and word fluency), learning and delayed recall, attention (target detection, serial sevens subtraction, and forward and backward digits), language (sentence repetition and verbal fluency), and orientation to time and place [36].

The TOVA (TOVA Company, Los Alamitos, CA, USA [37]) was used as an objective, computer-based series of tests that measure the attention and speed of processing to visual stimuli [38]. In the developed world, these measurements are compared to previously established norms; however, we used the HIV-negative group as the source of norms for the test. The TOVA has several advantages, including the use of visual stimuli, measurement of response times precisely (±1 millisecond), is language- and culture-free, and has a history of use in resource-challenged areas [38]. The visual component was used for this study, since this complements the auditory results from CATs. This attention component was important because the CATs (GAP, HINT, TDT) require sustained attention, and poor performance on these tests could be due to either difficulty in processing sound or a general difficulty in maintaining attention. Individuals who have difficulty with both the visual component of the TOVA as well as CATs may have a more generalized cognitive dysfunction related to processing speed or attention. For this project, we used the total mean response time (to the correct responses across the entire test), total exponentially modified Gaussian (ExGaussian) *μ* (mean response time of the correct responses modeled using the ExGaussian distribution), and attention comparison score (a composite score comparing the subject’s performance to a study of independent individuals diagnosed with attention deficit hyperactive disorder). Mean response time and ExGaussian *μ* were chosen to provide a direct measure of speed of processing. Particularly, ExGaussian *μ* provides a more precise distribution of response times to better assess processing speed [39,40]. Response times do not follow a normal Gaussian distribution due to factors such as fatigue and sequential effects [41]. Instead, response time distributions rise rapidly after stimulus presentation and have a long positive tail. This type of distribution is similar to the ExGaussian distribution [42], which is a mixture of a Gaussian and an exponential distribution that has been shown to fit response time distributions accurately [40]. The attention comparison score was chosen as a broad measure of inattention and impulsivity related to response time, omission error, and commission errors. Adults suffering from attention deficit hyperactive disorder or other cognitive disorders generally show variable processing speed with increased inattention (omission errors) or impulsivity (commission errors) [43].

The final cognitive test battery, the Cogstate battery [44], was chosen because it uses culturally neutral stimuli (eg, playing cards) to ensure that the assessment is not limited by a participant’s level of education. Card games are popular in Tanzania, and therefore the card-playing approach was familiar to the cohort. The Cogstate tasks are computer-based and designed for repeated administration. The Cogstate battery has been used to assess cognitive function in patients with HIV and has been shown to correlate well with standard neuropsychological test batteries [45-48]. For this project, we used the tests for visual learning and memory (One Card Learning Task, Continuous Paired Associate Learning Task, and Groton Maze Learning Test-with Delayed Recall) and attention/working memory (One Back Test). The One Back test also assessed processing speed. These tests were chosen to assess a broad range of executive functioning related to latent cognitive decline in HIV [32,38,46] and central auditory processing [10,17,18].

### Study Groups

To test the applicability of CATs to track cognitive function over time, we created four experimental groups. The first group consisted of HIV-negative individuals. We used this group to create normative values for each central auditory and cognitive test. To divide the HIV-positive group on the basis of CAT performance, we used a combination of transformed *z*-scores (using the scores of HIV-negative subjects as the standard) from the three CAT measures. That is, the HIV-negative group served as a normative reference for CAT performance for the HIV-positive group. Using the three CAT measures also ensured that those with poor central auditory function were identified accurately and not misclassified based on an outlier from an individual test. This combination score was based on the CAT results from the last visit (ie, the latest visit over the course of 3.5 years). The last visit was chosen since this should yield the greatest reductions in cognitive function due to time. We also noticed a learning effect across both cognitive tests and CATs over the course of the study (ie, scores improved over time). Therefore, identifying CATs at the last visit helped to mitigate this learning effect in the data analysis. In other words, by the last visit, subjects would have had ample time to learn the test; therefore, if the test results were still poor, we could interpret that as a deficit in central auditory processing.

Three groups were created based on the combination CAT score. One group included HIV-positive individuals whose performance on the GAP, HINT, and TDT combination *z*-score was in the top 20% (0.80 quantile, designated “TopCATs”) and the other group included HIV-positive individuals in the bottom 20% (0.20 quantile, designated “BottomCATs”) of the entire cohort at the time of their last visit. We hypothesized that those in the bottom 20% at the time of their last visit would be subjects with poor central auditory processing and cognitive function. The final group was created by simply taking all of the HIV-positive individuals that did not qualify in the TopCATs or BottomCATs category (HIV-positive group). This preliminary analysis resulted in four study groups: (1) HIV-negative, (2) HIV-positive, (3) TopCATs, and (4) BottomCATs.

### Statistical Analysis

Analyses were conducted using linear mixed-effects models with MATLAB 2020a (Mathworks, Natick, MA). Response variables included measures from the TOVA, Cogstate, and total score on the MoCA. Fixed effects included group (HIV-negative, HIV-positive, TopCATs, BottomCATs), age at last visit, and time between tests. Random effects included individual subject result variation over time. Using age and time as fixed effects allowed for analyses of cross-sectional age differences between subjects and longitudinal changes within subjects across time. This approach was developed by Laird and Ware [49] to study longitudinal epidemiological changes [50] and even changes in hearing loss [51].

We calculated *z*-scores for individual TOVA variables and Cogstate subtests using the HIV-negative cohort as the reference sample. We then calculated global scores of executive function, speed of processing, and central auditory scores from the *z*-scores using an approach similar to those proposed by Kamminga et al [48] and De Francesco et al [52]. These global scores allowed us to better understand the key domains of cognition, executive function, and speed of processing. The global executive score was calculated by combining all of the Cogstate subtest scores except the One Back Test (subtest of attention and processing speed) into a single variable of cognitive function. The global speed score was calculated for the TOVA subtests and One Back Test. The global hearing score was calculated from the GAP, HINT, and TDT. The primary hypothesis testing focused on the difference in the longitudinal change of cognitive variables between groups (interaction of time and group with age included in the model), specifically between the HIV-positive and BottomCATs groups, to better understand and track those with developing cognitive dysfunction due to HIV.

## Results

Table 1 shows the demographic characteristics of the overall cohort and for each group. BottomCATs were significantly different in age and pure tone average (PTA; average audiometric thresholds of 0.5, 1.0, 2.0, and 4 kHz) from the other groups. The BottomCATs group was about 1.3 years older than the HIV-positive group. Although all audiometric thresholds were <25 dB HL for all groups, the BottomCATs group showed worse PTAs in both ears (about 1.15 dB in each ear) compared to the HIV-positive group. Years of education did not significantly differ between the HIV-positive and BottomCATs groups. However, MoCA scores were significantly different between these two groups. In general, the HIV-negative group was about 15 years younger than the HIV-positive group, and comprised more men than the other groups. TopCATs were 4 years younger and comprised more men compared with the BottomCATs. Years of education was also significantly different between TopCATs and BottomCATs (with about 1.1 more years of education in the TopCATs), and between the HIV-negative and HIV-positive groups (the HIV-negative group had about 1.4 more years of education).

**Table 1:** Demographic information.

Characteristic		Overall Cohort (N=382)	HIV-negative (n=90)	HIV-positive (n=164)	TopCATs^a^ (n=53)	BottomCATs^b^ (n=75)	*P* value^c^		
							HIV-negative vs HIV-positive	TopCATs vs BottomCATs	HIV-positive vs BottomCATs
**Gender, n (%)**									
	Male	130 (34.0)	44 (49)	44 (26.8)	32 (59)	17 (23)	N/A^d^	N/A	N/A
	Female	239 (62.7)	46 (51)	119 (72.6)	21 (41)	58 (77)	N/A	N/A	N/A
Age (years), mean (SD)		37.8 (14.8)	25.9 (11.8)	40.8 (13.6)	38.1 (12.8)	42.1 (8.4)	<.001	.001	.001
**PTA^e^, mean (SD)**									
	Right ear	7.62 (6.1)	3.93 (6.8)	7.25 (5.2)	5.06 (5.2)	8.42 (8.2)	<.001	.001	.02
	Left ear	6.43 (6.6)	3.53 (7.2)	6.87 (5.9)	5.88 (5.0)	7.29 (6.1)	<.001	.001	.03
Education (years), mean (SD)		9.01 (2.7)	10.23 (2.6)	8.83 (2.6)	9.7 (2.3)	8.62 (2.8)	<.001	.001	.34
MoCA^f^, mean (SD)		27.6 (3.0)	28.2 (3.4)	27.2 (3.1)	27.9 (2.8)	26.7 (3.3)	.009	.01	.04

^a^TopCATs: in the top 20% of central auditory test results for HIV-positive individuals.

^b^BottomCATs: in the bottom 20% of central auditory test results for HIV-positive individuals.

^c^Based on two-sample *t* tests.

^d^N/A: not applicable.

^e^PTA: pure tone average (0.5, 1.0, 2.0, 4.0 kHz).

^f^MoCA: Montreal Cognitive Assessment.

**Figure 1:**
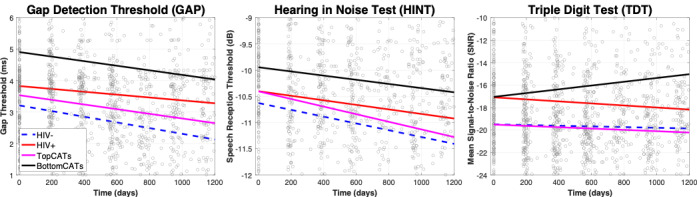
Trajectory of central auditory tests over time. Time 0 is the first visit in the study. Subjects were tested at roughly 6-month intervals thereafter. The blue dashed line shows the slope of the HIV-negative group, the red line shows the slope of the HIV-positive group, the magenta line shows the slope of the TopCATs group, and the black line shows the slope of the BottomCATs group. GAP, HINT, and TDT scores were used in combination to create the TopCATs and BottomCATs groups. The lines were fit to the data using linear fitting procedure in MATLAB. CAT: central auditory test; TopCATs: HIV-positive individuals in the top 20% of CAT scores; BottomCATs: HIV-positive individuals in the bottom 20% of CAT scores.

**Figure 2:**
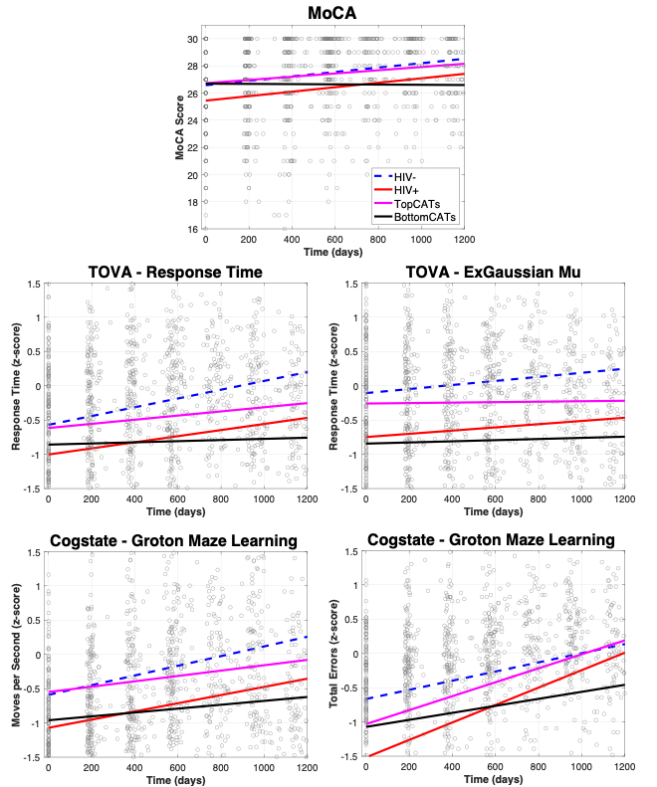
Trajectory of MoCA, TOVA, and Cogstate measures over time. The color scheme of lines for each group is the same as that in Figure 1. Cognitive measures of the TOVA and Cogstate were all transformed to z-scores using the HIV-negative group as normative values (the MoCA was not transformed). MoCA: Montreal Cognitive Assessment; TOVA: Test of Variables of Attention.TopCATs: HIV-positive individuals in the top 20% of central auditory test scores; BottomCATs: HIV-positive individuals in the bottom 20% of central auditory test scores.

**Figure 3:**
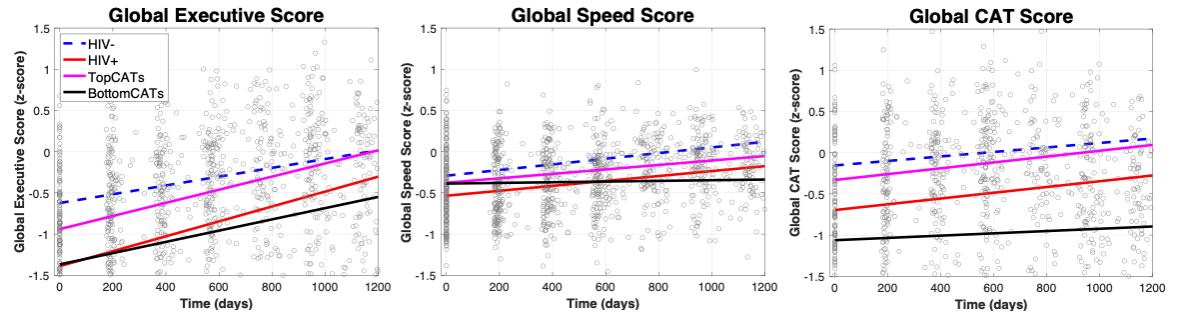
Trajectory of derived global scores over time. The color scheme of lines for each group is the same as that in Figure 1. CAT: central auditory test; TopCATs: HIV-positive individuals in the top 20% of central auditory test scores; BottomCATs: HIV-positive individuals in the bottom 20% of central auditory test scores.

Table 2 shows the results of the linear mixed models examining the β estimate of time (ie, slope), interaction of age and group, and interaction of time and group on CATs and cognitive variables of the MoCA, TOVA, Cogstate, and global scores. The comparison of HIV-positive and BottomCATs over time (time×group interaction) was of interest to analyze the longitudinal change in cognitive variables over time. HIV-positive was selected as the reference variable and HIV-negative was omitted from the model results, as the HIV-negative group was significantly younger than the HIV-positive group. The main effects of age and time are also omitted in the table (although age was included in the model) as they generally showed significant effects across all models due to the age difference in the HIV-negative group (younger HIV-negative subjects generally performed better over time). The interaction of age and group was of interest due to the significant difference between the HIV-positive and BottomCATs groups. That is, if the interaction of age and group was significant between the HIV-positive and BottomCATs groups, this effect could have mediated the interaction of time and group. Results of the interaction of age and group showed no significant differences between the HIV-positive and BottomCATs groups across all experimental variables, although the TOVA response time did approach significance (*P*=.057). Overall, the results are consistent with BottomCATs displaying a significant difference in trajectory over time compared to the HIV-positive group (interaction of time and group) in multiple cognitive subsets and derived global scores. Individual *P* values indicating a difference in slope are shown in Table 2 (β estimate of time) and are discussed below for CATs (Figure 1), cognitive subtests (Figure 2), and global scores (Figure 3).

**Table 2:** Results of linear mixed effect models.

Variable		Time estimate (slope, *β*)			Age×group *P* value			Time×group *P* value		
		HIV+	Top^a^	Bottom^b^	HIV+	Top	Bottom	HIV+	Top	Bottom
**CATs^c^**										
	Gap detection threshold (ms)	–4.22 ×10^–4^	–7.01 ×10^–4^	–5.33×10^–4^	Ref^d^	.54	.45	Ref	.03	.43
	HINT^e^ (SRT^f^)	–4.68×10^–4^	–5.61 ×10^–4^	–4.10×10^–4^	Ref	.53	.16	Ref	.17	.89
	TDT^g^ (mean SNR^h^)	–8.93×10^–4^	–9.01×10^–4^	2.16×10^–4^	Ref	.32	.92	Ref	.99	<.001
MoCA^i^ (total score)		15.5×10^–4^	12.5×10^–4^	–1.96×10^–4^	Ref	.19	.99	Ref	.56	.003
**TOVA^j^**										
	Response time (mean)	5.70×10^–4^	4.91×10^–4^	2.19×10^–4^	Ref	.63	.06	Ref	.47	.007
	ExGaussian^k^ *μ* (mean response time)	3.55×10^–4^	1.01×10^–4^	0.942×10^–4^	Ref	.47	.23	Ref	.08	.048
	Attention comparison score	4.50×10^–4^	3.42×10^–4^	4.08×10^–4^	Ref	.72	.29	Ref	.09	.13
**Cogstate battery tests**										
	Groton maze learning (moves per second)	1.05×10^–4^	0.721×10^–4^	0.511×10^–4^	Ref	.84	.69	Ref	.18	.046
	Groton maze learning (total errors)	13.8×10^–4^	11.3×10^–4^	6.15×10^–4^	Ref	.24	.16	Ref	.45	.03
	One Card Learning (accuracy)	3.86×10^-4^	2.95×10^-4^	0.761×10^-4^	Ref	>.99	.71	Ref	.78	.08
	One Back Test (reaction time)	13.2×10^-4^	9.12×10^-4^	10.9×10^-4^	Ref	.62	.18	Ref	.06	.16
	Continuous paired associate learning (accuracy)	4.44×10^-4^	2.98×10^-4^	4.05×10^-4^	Ref	.28	.08	Ref	.16	.07
Global executive score^l^		9.61×10^-4^	8.62×10^-4^	7.31×10^-4^	Ref	.47	.21	Ref	.30	.02
Global speed score^m^		3.04×10^-4^	3.00×10^-4^	0.511×10^-4^	Ref	.35	.07	Ref	.89	.006
Global CAT score		3.59×10^-4^	4.21×10^-4^	0.540×10^-4^	Ref	.45	.23	Ref	.58	.01

^a^Top: HIV-positive individuals in the top 20% of combined central auditory test scores.

^b^Bottom: HIV-positive individuals in the bottom 20% of combined central auditory test scores.

^c^CAT: central auditory test.

^d^Reference for comparison.

^e^HINT: Hearing In Noise Test.

^f^SRT: speech reception threshold.

^g^TDT: Triple Digit Test.

^h^SNR: signal-to-noise ratio.

^i^MoCA: Montreal Cognitive Assessment.

^j^TOVA: Tests of Variables of Attention.

^k^ExGaussian: exponentially modified Gaussian distribution.

^l^Combination of speed-of-processing subtests.

^m^Combination of executive functioning subtests.

Figure 1 shows the GAP, HINT, and TDT scores over time for each experimental group. Overall, the BottomCATs group showed poorer scores over time compared to all other groups. BottomCATs showed similar parallel trajectories in the GAP and HINT compared to the HIV-positive group. The TDT showed a significantly worse trajectory in the BottomCATs group compared to that of the HIV-positive group. This is consistent with BottomCATs neutralizing the learning effect seen across variables, exhibiting worsening scores over time.

Figure 2 shows the significant MoCA, TOVA, and Cogstate trajectories over time for each experimental group. Overall, these results are consistent with a trend of BottomCATs displaying a difference in trajectory of cognitive variables over time. However, the Attention Comparison Score, Continuous Paired Associate Learning, One Back Test, and One Card Learning trajectories were not significantly different from those of the HIV-positive group (Table 2). Trajectories showed an overall improvement in scores in the HIV-negative, HIV-positive, and TopCATs groups, presumably due to a learning effect. Interestingly, the BottomCATs group exhibited a nearly flat trajectory over time and showed significantly different slopes compared to those of the HIV-positive group on the MoCA, TOVA response time, and ExGaussian *μ* (Table 2). The Cogstate Groton Maze Learning subtest showed similar results, displaying a significant difference in trajectory between HIV-positive and BottomCATs in moves per second and total errors (Table 2). That is, HIV-positive individuals who scored in the bottom 20% of CATs at the time of their last visit showed a difference in trajectory of cognitive results over time compared to the overall HIV-positive group in measures of both executive function (Cogstate Groton Maze Learning) and speech of processing (TOVA response time and ExGaussian *μ*).

Figure 3 shows the global scores derived from the TOVA and Cogstate subtests. Each global score showed a difference in trajectory between the HIV-positive and BottomCATs groups in the global executive score (derived from Cogstate subtests), global speed score (derived from TOVA subtests and One Back subtest from Cogstate), and global CAT score (derived from the GAP, HINT, and TDT) (Table 2). Particularly, the global speed score showed a nearly flat trajectory over time, which was similar to the MoCA trajectories.

## Discussion

### Principal Results

Among HIV-positive individuals, the trajectory of cognitive performance over time differed as a function of CAT performance. Over the time period studied, those individuals with HIV that fell in the bottom 20% on a combination of CATs either worsened or improved more slowly than the other groups. In contrast, both HIV-negative patients and HIV-positive patients with good CAT performance improved in their cognitive performance, potentially reflecting learning effects on the tasks. This suggests that CAT scores in those diagnosed with HIV are correlated with a worsening trajectory or failure to improve on measures of cognitive function.

Results from this study suggest that CATs may be a useful way to provide surveillance for the development of neurocognitive problems in people with HIV. Previous studies have suggested that HIV-positive individuals develop signs of central auditory processing deficits [8,33,53,54]. These deficits could reflect CNS damage from HIV infection or treatment. Even with advancing antiretroviral therapy, HIV-positive individuals may potentially develop neurocognitive deficits [1]. We aimed to build on this premise and have provided longitudinal evidence relating performance on a battery of three CATs to cognitive function in HIV-positive individuals. This study is the first to show that HIV-positive individuals with poor CAT performance (despite normal peripheral hearing) have a worse trajectory or fail to improve on measures of cognitive function over time compared with HIV-positive individuals with typical CAT scores. Since this is occurring despite otherwise normal hearing sensitivity up to 4.0 kHz, the changes in cognitive function over time may be explained by deficits in central auditory processing.

Results of this study also suggest that the main problems of CATs are in the domains of executive function and processing speed. This is evidenced by significant results on the MoCA, TOVA, Cogstate, and global scores. The MoCA displayed the strongest interaction (*P*=.003) and also showed a negative trajectory over time (slope –1.96×10^-4^) for the BottomCATs group. The MoCA has been shown to be a relatively sensitive measure of cognitive dysfunction in HIV-positive individuals [55,56]. TOVA and Cogstate subtests also showed a significant difference in the trajectory of cognitive variables over time, but one TOVA subtest (Attention Comparison Score) and three Cogstate subtests (Continuous Paired Associative Learning, One Back Test, One Card Learning) did not show the same pattern. This could have been due to various factors such as variability of the subtests, elevated learning effects, or that these subtests are not sensitive to cognitive dysfunction in the experimental HIV cohort. By contrast, global scores, which included these nonsignificant variables, resulted in significant differences in trajectories over time. With the combination of speed and executive variables in global scores (global executive, global speed), the correspondence of cognitive performance to BottomCATs as an indicator of neurocognitive dysfunction became more robust. These global scores emerged as strong between-group trajectory differences between the HIV-positive and BottomCATs groups beyond the individual variability, to which singular cognitive measures are particularly susceptible. The combination of MoCA, TOVA, Cogstate, and global scores further supports the interpretation that CATs are sensitive in the detection of a cognitive dysfunction in those diagnosed with HIV.

HIV-positive individuals with poor central auditory function also showed degradations in processing speed on a variety of cognitive subtests over time. For example, the mean response time and ExGaussian *μ* on the TOVA, moves per second on the Groton Maze Learning, and the global speed score showed significant differences in trajectory of those with poor CAT scores over time. Although age could undoubtedly have an effect on processing speed, the interaction between age and group was not significant in any of the linear mixed effect models. Processing auditory information quickly is essential for accurate communication, and the link between cognitive processing speed and CATs has been extensively studied [57-59]. Speech perception, specifically in background noise, places a substantial burden on processing speed, attention, and working memory. Unlike written text, speech processing is carried out in real time, with words coming in at a rapid rate of 120 to 180 words per minute, without opportunity for the listener to go back and review previous material [18]. In background noise, this complex process places even more demands on cognitive processing speed.

One interpretation of our results could be that some individuals with HIV experience an accelerated aging process. That is, HIV could be associated with accelerated cognitive aging such that a subset of people with HIV in their 40s and 50s are functioning with a cognitive processing speed typical of that found in people in their 60s and 70s. Cognitive and central auditory deficits in adults with HIV may result from additive effects of the pathophysiological mechanisms of aging (ie, the “common cause” hypothesis [60]) and HIV [61]. Previous longitudinal studies on HIV have shown significant interaction effects of HIV and age [62,63], suggesting that these mechanisms may be associated. For example, Seider et al [51] showed that older adults with HIV exhibited significant memory decline over the course of 1 year, but no decline was seen in younger adults with HIV or in HIV-negative controls regardless of age. Although problems with learning and memory are more typically reported in those with HIV [1,32], previous studies have also found deficits in processing speed [64-66]. If we interpret subjects in the BottomCATs group as those with accelerated aging, then the results are consistent with the aging and central auditory processing literature [57,67,68]. For example, robust effects of age on auditory temporal processing have been revealed when using complex tasks or stimuli [57,69]. Furthermore, robust age-related differences in gap detection have been observed when the markers surrounding a silent gap are shorter than 10 milliseconds [70] and when the location of the gap falls near the onset or offset of the stimuli, or is varied randomly [71]. These previous results in combination with this study suggest that accelerated aging due to HIV as evidenced by degraded cognitive speed-of-processing tests may be revealed by central auditory processing.

Future studies should seek to examine executive functioning and speed of processing in the central auditory pathway in HIV-positive individuals to improve upon surveillance of this public health concern. Declines in general cognitive processing speed have been considered a hallmark of the aging process, beginning in young adulthood and continuing nearly linearly across the lifespan [58]. However, it may be that the process in HIV-positive individuals has an altered slope or is nonlinear. It may also be that changes to neurocognitive function—and thus central auditory processing—are attributed to changes in earlier or automatic levels of processing, causing a cascade of degradations along the pathway. Neuroimaging research has confirmed age differences in brain activity related to processing speed, particularly in areas of the prefrontal cortex [72], as well as hearing-related differences in patterns of brain activation [73]. The exact links remain to be determined, but such structural and functional changes provide a mechanism for linking sensory and cognitive changes with age and HIV [54]. Finally, a more difficult central auditory task may be more sensitive to detecting HIV-related neurocognitive disorders with age. Although the CATs used in this study are not simple, adults with HIV may compensate for declines in early stages of auditory processing by exerting increased cognitive control or attention [74,75]. Consistent with this hypothesis, Alain et al [63] observed age-related differences in event-related potential amplitudes during passive listening in a simple gap detection task, but not with active listening, which may reflect a decline in automatic processing of temporally modulated stimuli compensated by attentional processes [63].

### Limitations

This study has limitations. The main limitation in interpreting the cognitive variables accurately over time was an overall learning effect. Although previous results have shown minor learning effects on these cognitive variables [76,77], we observed an overall trend for cognitive scores to improve over time. This could have been due to two factors. First, the subjects were learning how to execute the tests more accurately every time they came in for a visit. Even though 6 months between visits could be considered a long enough time to limit learning effects [9,78], we observed a general improvement over time in all cognitive tests. Second, the test administrators may have improved at conducting the tests. As stated previously, cognitive testing typically requires trained personnel to administer the tests. Although the test administrators were well trained at the onset of the study, it is possible that they became more proficient in explaining and instructing the cognitive tests over time. Nevertheless, the data show that examining the trajectory of cognitive change over time is important, rather than cross-sectional analysis.

Another limitation is that this study was conducted over a 3.5-year period, which is not an exceptionally long time to develop cognitive decline due to HIV. These data were from an ongoing project in Dar es Salaam, Tanzania. More time is needed for more individuals to complete multiple visits and for deterioration in neurocognitive performance to develop to fully answer the question of whether CATs can predict future cognitive decline. This study was only able to show the association.

Differences in PTA may have affected the results. Although normal hearing sensitivity from 0.5 to 4.0 kHz was required for inclusion, the difference in hearing thresholds (ie, the difference in PTA between the BottomCATs and HIV-positive groups) might have affected the CAT results. Although it is unlikely that an averaged difference for both ears of 1.2 dB in PTA affected the results, it is possible that peripheral hearing sensitivity also factored into the trajectory of cognitive variables over time. This could, however, also be related to damage not reflected in hearing thresholds, such as damage to the synapses between hair cells and the cochlear nerve or further along the auditory pathway. Studies have suggested that peripheral hearing sensitivity is not a comprehensive picture of auditory function [79-81].

### Conclusions

The overall results from this study suggest that CATs may be useful to track cognitive function over time in people with HIV. This could provide an easy-to-use, quick method of surveillance for this important public health problem. Subjects that performed in the bottom 20% of a battery of three CATs had a significantly different trajectory of cognitive variables over time, suggestive of cognitive dysfunction. The cognitive dysfunction seen was consistent with a failure to improve or decrease in executive functioning and speed of processing in those with poor central auditory function over time. This study supports the ideal that CATs should be studied further to track cognitive dysfunction in those with HIV-related cognitive deficits.

## Abbreviations

CAT: central auditory test
CNS: central nervous system
ExGaussian: exponentially modified Gaussian distribution
GAP: gap detection threshold
HINT: Hearing In Noise Test
MoCA: Montreal Cognitive Assessment
PTA: pure tone average
SNR: signal-to-noise ratio
TDT: Triple Digit Test
TOVA: Tests of Variables of Attention
WHATS: wireless automated hearing system
